# Gender-, Age- and Region-Specific Characterization of Vertebral Bone Microstructure Through Automated Segmentation and 3D Texture Analysis of Routine Abdominal CT

**DOI:** 10.3389/fendo.2021.792760

**Published:** 2022-01-27

**Authors:** Michael Dieckmeyer, Nico Sollmann, Malek El Husseini, Anjany Sekuboyina, Maximilian T. Löffler, Claus Zimmer, Jan S. Kirschke, Karupppasamy Subburaj, Thomas Baum

**Affiliations:** ^1^ Department of Diagnostic and Interventional Neuroradiology, School of Medicine, Klinikum rechts der Isar, Technical University of Munich, Munich, Germany; ^2^ TUM-Neuroimaging Center, Klinikum rechts der Isar, Technical University of Munich, Munich, Germany; ^3^ Department of Diagnostic and Interventional Radiology, University Hospital Ulm, Ulm, Germany; ^4^ Department of Radiology, University Medical Center, Albert-Ludwigs-University Freiburg, Freiburg, Germany; ^5^ Pillar of Engineering Product Development, Singapore University of Technology and Design, Singapore, Singapore; ^6^ Changi General Hospital, Singapore, Singapore

**Keywords:** automated segmentation, texture analysis, multi-detector computed tomography, osteoporosis, bone microstructure

## Abstract

**Purpose:**

To identify long-term reproducible texture features (TFs) of spinal computed tomography (CT), and characterize variations with regard to gender, age and vertebral level using our automated quantification framework.

**Methods:**

We performed texture analysis (TA) on baseline and follow-up CT (follow-up duration: 30–90 days) of 21 subjects (8 females, 13 males, age at baseline 61.2 ± 9.2 years) to determine long-term reproducibility. TFs with a long-term reproducibility error Δ_rel_<5% were further analyzed for an association with age and vertebral level in a cohort of 376 patients (129 females, 247 males, age 62.5 ± 9.2 years). Automated analysis comprised labeling and segmentation of vertebrae into subregions using a convolutional neural network, calculation of volumetric bone mineral density (vBMD) with asynchronous calibration and TF extraction. Variance_global_ measures the spread of the gray-level distribution in an image while Entropy reflects the uniformity of gray-levels. Short-run emphasis (SRE), Long-run emphasis (LRE), Run-length non-uniformity (RLN) and Run percentage (RP) contain information on consecutive voxels of a particular grey-level, or grey-level range, in a particular direction. Long runs (LRE) represent coarse texture while short runs (SRE) represent fine texture. RLN reflects similarities in the length of runs while RP reflects distribution and homogeneity of runs with a specific direction.

**Results:**

Six of the 24 extracted TFs had Δ_rel_<5% (Variance_global_, Entropy, SRE, LRE, RLN, RP), and were analyzed further in 4716 thoracolumbar vertebrae. Five TFs (Variance_global_,SRE,LRE, RLN,RP) showed a significant difference between genders (p<0.001), potentially being caused by a finer and more directional vertebral trabecular microstructure in females compared to males. Variance_global_ and Entropy showed a significant increase from the thoracic to the lumbar spine (p<0.001), indicating a higher degree and earlier initiation of trabecular microstructure deterioration at lower spinal levels. The four higher-order TFs showed significant variations between spine regions without a clear directional gradient (p ≤ 0.001-0.012). No TF showed a clear age dependence. vBMD differed significantly between genders, age groups and spine regions (p ≤ 0.001–0.002).

**Conclusion:**

Long-term reproducible CT-based TFs of the thoracolumbar spine were established and characterized in a predominantly older adult study population. The gender-, age- and vertebral-level-specific values may serve as foundation for osteoporosis diagnostics and facilitate future studies investigating vertebral microstructure.

## Introduction

Texture analysis (TA) is an emerging subfield of radiomics, representing a non-invasive and objective method for the quantitative assessment of medical images. Texture features (TFs) can be used to quantitatively characterize image properties, such as uniformity, heterogeneity and randomness, as well as repetitive image patterns. TA has the potential to enable the extraction of additional diagnostic, predictive and prognostic information beyond what is visually perceptive (1). Traditionally, applications of TA include neuroimaging, musculoskeletal and oncological imaging, e.g. to assess tumor heterogeneity (2, 3). TA has been performed across different anatomical regions and modalities, with CT being the modality used most frequently, arguably due to its multitude of clinical applications, broad availability and data quality (4). Overall, musculoskeletal applications have been based on radiographic, magnetic resonance imaging (MRI) (5, 6) and CT data (7–9).

Osteoporosis is a systemic disorder of bone metabolism characterized by reduced bone mineralization and microarchitectural deterioration of osseous tissue (10, 11). Bone health and the closely associated vertebral fracture (VF) risk are dependent on bone mineral density (BMD) and bone microstructure which is primarily defined by the three-dimensional (3D) trabecular bone architecture as suggested by high correlations with micro-CT as reference (12, 13). Volumetric BMD (vBMD) can be measured on a vertebra-specific level with high spatial resolution using quantitative CT (QCT), while CT-based TA enables the evaluation of trabecular bone microstructure. Therefore, osteoporosis represents one of the most promising clinical applications of TA. In a small study cohort, Mookiah et al. showed that TA is feasible for opportunistic osteoporosis screening and can discriminate subjects with and without VFs accurately while exhibiting acceptable long-term scan-rescan reproducibility. However, the size of the used study population does not enable the derivation of TF reference values (7). Muehlematter et al. used machine learning algorithms on CT-based TA to better predict incident VFs (8). Through the combination of 3D-TA and regional vBMD, Valentinitsch et al. could improve the classification of prevalent VFs. In this study, the eventually analyzed TFs were selected from a variety of features, using an exponential search based on the Gini Index and subsequently used for classification of VFs which represent the most relevant clinical outcome of osteoporosis. However, the actual TF values were not reported (9). In spine imaging, commonly used TFs include first-order, second-order and higher-order statistical features, and reliable extraction of specific TFs was derived from sagittal reformations of up to 3 mm slice thickness in routine abdominal multi-detector CT (MDCT) scans with administration of intravenous contrast medium (IVCM) (7). However, TA for the evaluation of the osseous microstructure of the spine and ensuing bone health has not been standardized, yet.

One essential and time-consuming part of the TF extraction process is the segmentation to create individual regions of interest (ROIs) of the vertebral bodies, which has usually been performed manually on single slices. However, recent advancements in deep learning, in particular in convolutional neural networks, enable a standardized 3D segmentation by a fully automated pipeline (14–16). In order to discriminate TF values indicative of osteoporosis from normal values, and to enable longitudinal analyses of vertebral microstructure, the characterization of reproducible TF values is needed and can therefore be considered a prerequisite for diagnostic and therapeutic decisions in osteoporosis that use TFs.

Therefore, purposes of our study were (i), to identify CT-based TFs of vertebral bone with high long-term reproducibility which would be particularly important for longitudinal studies in the context of intervention monitoring, and (ii), to generate an automated standardized pipeline for segmentation and TA of the spine to establish characteristic TF values and determine physiological variations of the identified highly reproducible TFs with regard to gender, age and vertebral level in a mainly older adult population.

## Materials and Methods

### Subjects

First, for the assessment of long-term reproducibility, a cohort of 21 Caucasian patients who received baseline (BL) and follow-up (FU) routine abdominal MDCT scans with IVCM and a FU duration between 30 and 90 days were identified. Clinical indications were oncologic staging for the BL scans, and ruling out postoperative complications (e.g. anastomotic insufficiencies, fistulas, infections) for the FU scans. For each TF and vertebra, the relative difference between BL and FU measurement Δ_rel_ was calculated as a measure of long-term reproducibility,


$$\Delta_{rel}=2\left|\frac{TF_{FU}-TF_{BL}}{TF_{FU}+TF_{BL}}\right|,$$

with *TF_FU_* and *TF_BL_* denoting the measurements of a certain TF at FU and BL, respectively, and averaged across all vertebrae (T4 - L5) for each patient. All TFs showing a reproducibility error Δ_rel_ < 5% were investigated further with regard to gender, age and vertebral level in a larger patient cohort as described in the following.

Second, 376 Caucasian patients who received routine abdominal MDCT scans with IVCM were analyzed. Clinical indication was oncologic follow-up to rule out tumor recurrence. For each of these patients, all vertebrae from T4 to L5, completely included in the field of view (FOV), were analyzed.

All patients were retrospectively identified in our hospital’s digital picture archiving and communication system (PACS). Inclusion criteria were the availability of an MDCT scan including the spine at the same scanner with a specific protocol as outlined below. Exclusion criteria were VFs, osteoporosis, history of bisphosphonate or other bone metabolism-influencing therapy, osseous metastases as well as hematological or other metabolic bone disorders. Osteoporotic vertebrae were defined as vBMD < 80 mg/cm^3^ according to the cutoff values suggested for spine QCT measurements by the American College of Radiology (ACR) (17). The present study was approved by the local institutional review board. The requirement of the written consent was waived due to the retrospective nature of the study.

### CT Image Acquisition

All CT data was acquired on the same 64-row MDCT scanner (Somatom Sensation Cardiac 64; Siemens Medical Solutions, Erlangen, Germany). A standardized protocol with the following dedicated scanning parameters was applied. Average tube voltage: 120 kVp, adapted tube load: 200 mAs (averaged), minimum collimation of 0.6 mm. An intravenous contrast agent (Imeron 400, Bracco, Konstanz, Germany) was administered for each examination following a standard protocol using a high-pressure injector (Fresenius Pilot C; Fresenius Kabi, Bad Homburg, Germany) with a delay of 70 s, a flow rate of 3 mL/s, and a body weight-dependent dose (80 mL for body weight ≤ 80 kg, 90 mL for body weight > 80 kg and ≤ 100 kg, and 100 mL for body weight > 100 kg). Oral contrast medium (1000 mL of Barilux Scan; Sanochemia Diagnostics, Neuss, Germany) was given before each CT scan. Sagittal reformations of the spine with a slice thickness of 3 mm and reconstructed with a standard bone kernel were used for TA.

### Automated Image Segmentation

**Figure 1:**
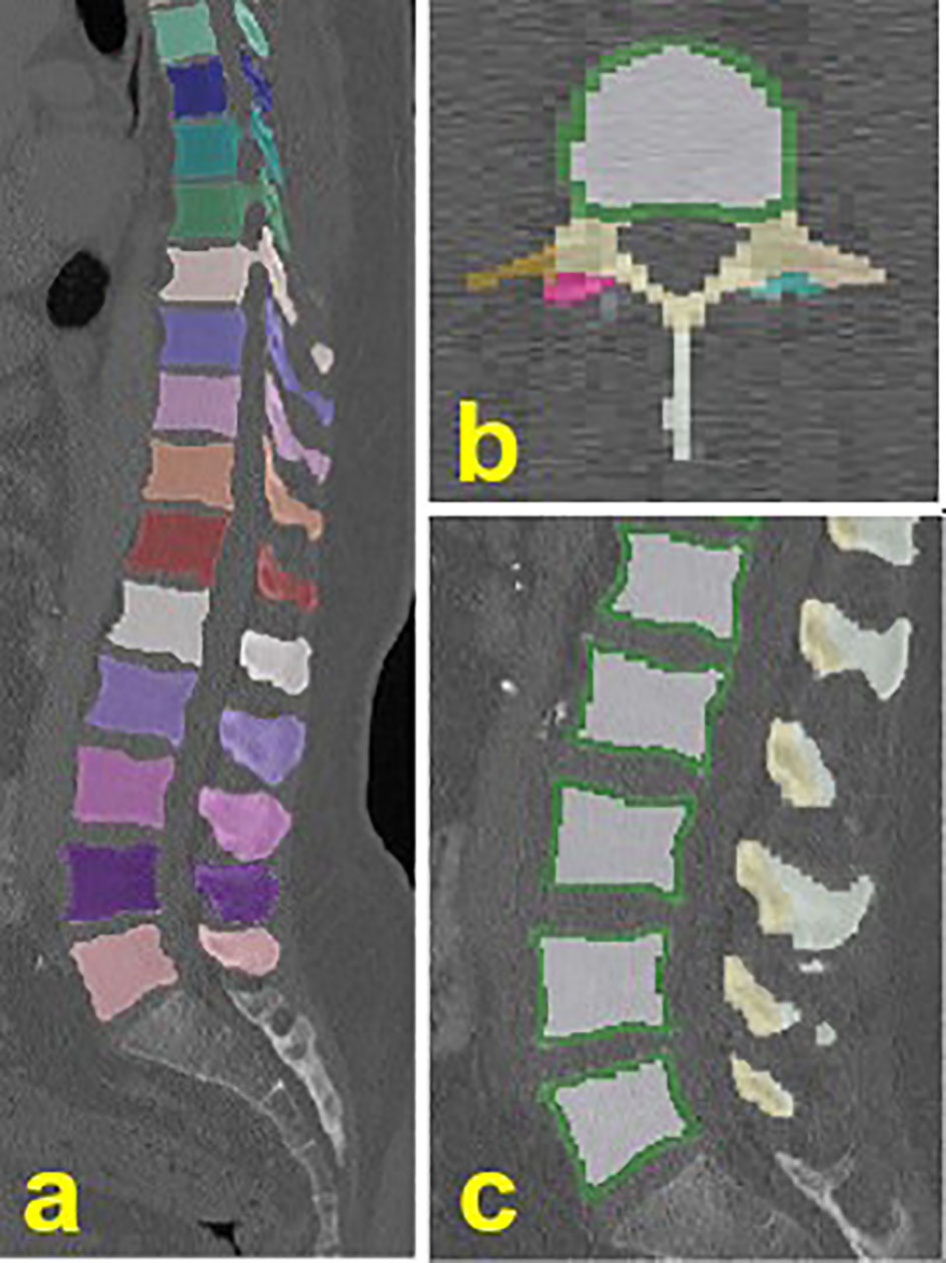
Exemplary illustration of the automated labeling and segmentation. **(A)** individually labeled and segmented vertebrae from T4-L5. **(B, C)** vertebral subregions, including the trabecular (white) and cortical (green) compartment of the vertebral body.

The vertebrae T4 to L5 were automatically segmented in the MDCT images using a deep learning-driven framework (https://anduin.bonescreen.de). The freely available tool identifies and labels each vertebra in a fully automated process and creates corresponding segmentation masks. Additionally, for each vertebra, a defined set of segmentation masks of subregions, including the trabecular compartment of the vertebral body among others, is created (**Figure 1**) (14, 15). The fully automatically generated labels and segmentation masks of all vertebrae were checked visually by a radiologist with two years of experience in spine imaging, and manually corrected if necessary. In total, labels and segmentations in 53 vertebral bodies of the 376 analyzed patients were manually corrected. Causes of imperfect labeling and segmentation were Schmorl nodes (33 vertebrae), severe degenerative changes (18), partial block vertebrae (3), hemivertebrae (1), or thoracolumbar and lumbosacral transitional vertebrae (7).

### Texture Analysis

All TFs were calculated for the ROI corresponding to the trabecular compartment of each segmented vertebral body. The extracted TFs included three global features, also referred to as first-order statistical moments, which are computed by gray-level histogram analysis, eight second-order features, based on gray-level co-occurrence matrix (GLCM) analysis, and 13 higher-order features, based on gray-level run-length matrix (GLRLM) analysis (**Table 1**). In total, 24 TFs were extracted which quantify textural patterns (e.g. fine, coarse, smooth, or irregular) in an image. Each GLCM entry represents the probability of gray-level co-occurrence between voxel pairs at a given vector direction and a fixed length of 1 between the voxel pair. Each GLRLM entry is the probability of voxel occurrences of a specific gray-level for a possible run-length. A gray-level run is a set of successive voxels with identical gray-level values arranged collinearly along a given vector direction, and the run length is defined as the number of voxels in it. GLRLM features are computed based on the occurrence and distribution of such runs in a given image and measure the directional changes in the GLCM (26). In total, there are 13 direction vectors with a displacement of (d*x, *d*y, *d*z*). The matrices of all 13 directions were averaged and normalized before calculating the second-order GLCM and higher-order GLRLM indices. GLCM is an (*n* × *n*) matrix where *n* is the number of gray-levels in each image. GLRLM is an (*n* × *m*) matrix, where *n* is the number of gray-levels in the scan, and *m* is the run length. **Table 1** gives an overview of the analyzed TFs together with descriptions of the quantified image properties.

**Table 1:** Global (histogram-based), gray-level co-occurrence matrix (GLCM)-based, and gray-level run-length matrix (GLRLM)-based texture features and descriptions.

Category	Texture feature	Description (What is quantified)?	Reference
Global	Variance	Spread of gray-level distribution	Gaztañaga et al. (20)
(Histogram)	Skewness	Shape of gray-level distribution	
	Kurtosis	Flatness of gray-level distribution	
Second-order	Energy	Uniformity	Haralick et al. (21)
(GLCM)	Contrast	Local intensity variation	
	Entropy	Randomness	
	Homogeneity	Homogeneous scene	
	Correlation	Linear spatial relationships between texture elements	
	Sum average	Spread of the mean voxel co-occurrence distribution	
	Variance	Voxel co-occurrence distribution	
	Dissimilarity	Heterogeneity	
Higher-order	SRE	Short run distribution	Galloway et al. (22)
(GLRLM)	LRE	Long run distribution	
	GLN	Similarities of gray-level	
	RLN	Similarities in length of runs	
** **	RP	Distribution and homogeneity of runs with a specific direction	
** **	LGLRE	Distribution of low-gray-level values	Chu et al. (23)
** **	HGLRE	Distribution of high-gray-level values	
** **	SRLGE	Joint distribution of short runs and low gray-level values	Dasarathy et al. (24)
** **	SRHGE	Joint distribution of short runs and high gray-level values	
** **	LRLGE	Joint distribution of long runs and low gray-level values	
** **	LRHGE	Joint distribution of long runs and high gray-level values	
** **	GLV	Weighted variances of gray-level values	Thibault et al. (25)
** **	RLV	Weighted variances of gray-level runs	

SRE, short-run emphasis; LRE, long-run emphasis; GLN, gray-level non-uniformity; RLN, run-length non-uniformity; RP, run percentage; LGLRE, low gray-level run emphasis; HGLRE, high gray-level run emphasis; SRLGLE, short-run low gray-level emphasis; SRHGLE, short-run high gray-level emphasis; LRLGLE, long-run low gray-level emphasis; LRHGLE, long-run high gray-level emphasis; GLV, gray-level variance; RLV, and run-length variance; table adapted from (19).

In order to generate isotropic volumes of the image datasets necessary for comparable TA results, cubic interpolation was used. To prevent sparseness, gray-level quantization was performed using the normalized gray-levels (scale 0 to 1) of the ROI corresponding to the trabecular compartment of each vertebral body. All steps of the TA were performed with MATLAB (version R2021a; MathWorks Inc., Natick, MA, USA) using a modified version of a publicly available radiomics toolbox (https://github.com/mvallieres/radiomics) (27).

### vBMD Extraction

vBMD was calculated for the ROI corresponding to the trabecular compartment of each segmented vertebral body applying asynchronous calibration to convert CT attenuation, measured in Hounsfield units (HU), to vBMD. Asynchronous calibration uses bone-equivalent density phantoms to generate HU-to-BMD conversion equations for a specific CT scanner and acquisition protocol. Previously established HU-to-BMD conversion equations were applied in this study using a phantom with hydroxyl-apatite inserts of known density in mg/cm³ (Anthropomorphic Abdomen Phantom, QRM Quality Assurance in Radiology and Medicine) (14). Linear correction equations for the presence of IVCM in portal-venous phase were applied to avoid vBMD bias (28).

### Statistical Analysis

Statistical analysis was performed with SPSS (SPSS Inc., Chicago, IL, USA) and MATLAB (version R2021a; The Mathworks, Natick, MA, USA) using a two-sided level of significance of 0.05 for all statistical tests. The Kolmogorov-Smirnov test indicated normally distributed data for the analyzed TFs and vBMD, irrespective of gender. The association between vBMD and the analyzed TFs was determined using Pearson correlation coefficient (*r*). Independent *t*-tests were used to test for significant gender-dependent differences of TFs and vBMD. Analysis of variance (ANOVA) was used to test for significant differences across age groups and vertebral levels, respectively. For all tests, Bonferroni correction was applied to adjust for multiple comparisons.

## Results

### Long-Term Reproducibility of TFs

For each of the 21 included patients (8 females, 13 males, mean age at BL = 61.2 ± 9.2 years, age range at BL = 43.7 – 71.6 years), T4 to L5 were analyzed, resulting in a total of 294 analyzed vertebrae. The mean follow-up duration, which is the time interval between the BL and FU scan, was 59 ± 11 days (range: 34 – 77 days). Of the 24 analyzed TFs, four features showed a relative difference between FU and BL measurements (Δ_rel_) below 1%, and two features between 1% and 5% (**Table 2**). In total, six TFs showed a reproducibility error Δ_rel_ < 5% (Variance_global_, Entropy, SRE, LRE, RLN, RP) and were further analyzed with regard to gender, age and vertebral level.

**Table 2:** Relative difference Δ_rel_ between baseline and follow-up measurement of the analyzed TFs.

**Texture feature**	**Δ_rel_ [%]**
Variance_global_*	2.22
Skewness_global_	248.63
Kurtosis_global_	76.45
Energy	45.16
Contrast	64.63
Entropy*	3.94
Homogeneity	45.34
Correlation	52.21
SumAverage	18.82
Variance	33.20
Dissimilarity	40.26
SRE**	0.15
LRE**	0.59
GLN	27.66
RLN**	0.38
RP**	0.19
LGLRE	9.38
HGLRE	21.57
SRLGLE	9.33
SRHGLE	21.64
LRLGLE	9.59
LRHGLE	21.32
GLV	34.64
RLV	34.32

TF, texture feature; SRE, short-run emphasis; LRE, long-run emphasis; GLN, gray-level non-uniformity; RLN, run-length non-uniformity; RP, run percentage; LGLRE, low gray-level run emphasis; HGLRE, high gray-level run emphasis; SRLGLE, short-run low gray-level emphasis; SRHGLE, short-run high gray-level emphasis; LRLGLE, long-run low gray-level emphasis; LRHGLE, long-run high gray-level emphasis; GLV, gray-level variance; RLV, run-length variance.

TFs with Δ_rel_ < 1% and Δ_rel_ < 5% are marked with ** and *, respectively.

### TF Variations With Regard to Gender, Age and Vertebral Level

**Figure 2:**
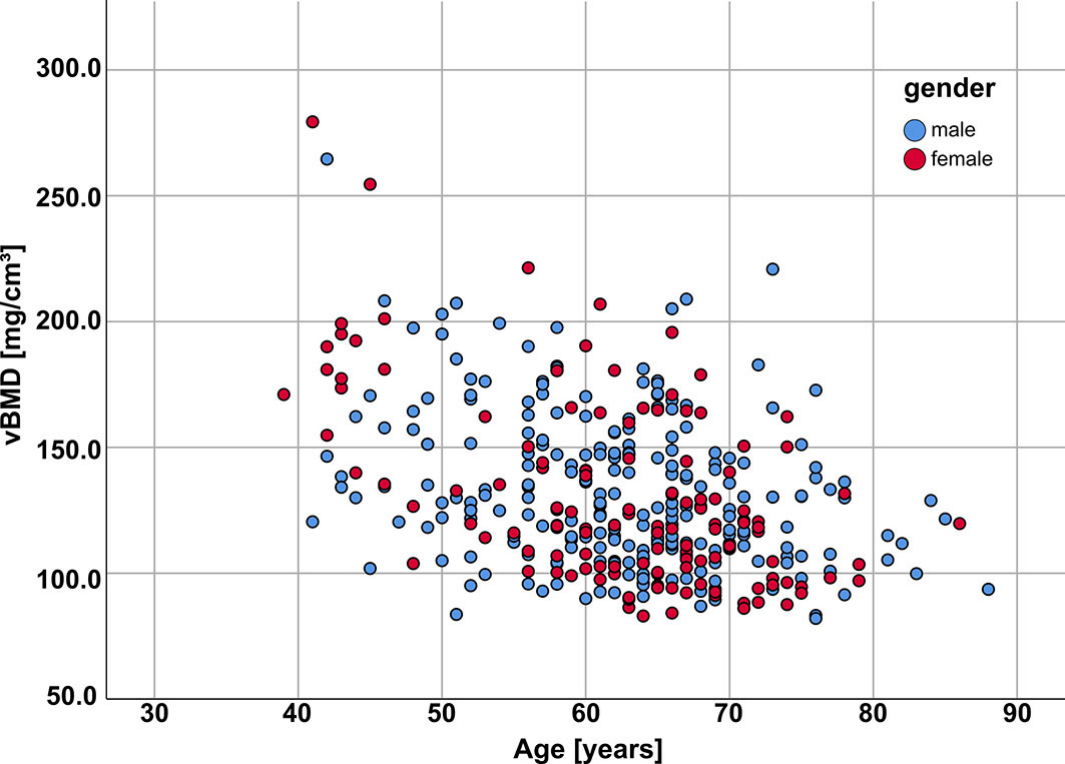
Patient age *vs* vBMD (averaged across all analyzed vertebrae for each patient) for females (red) and males (blue); vBMD, volumetric bone mineral density.

In total, 376 patients (129 females, 247 males, mean age = 62.5 ± 9.2 years, age range = 39.0 – 88.0 years) were included, and stratified into four age groups for each gender, respectively: <50 years, 50-59 years, 60-69 years, and ≥70 years (**Figure 2** and **Table 3**). In total, vertebral bodies from T4 to L5 were included. In total, labels and segmentations were manually corrected in 53 of the 4716 vertebral bodies, resulting in a correction ration of 1.1%. The analyzed vertebral bodies were stratified into three spine regions: mid thoracic spine (T4-T8), lower thoracic spine (T9-T12) and lumbar spine (L1-L5). TF values with regard to gender, age and vertebral level are summarized in **Tables 4**–**7**.

**Table 3:** Patient demographics stratified by age groups. Mean ± standard deviation (SD) for age (in years).

		Male	Female
< 50 years	*n*	21	17
	Age	45.9 ± 2.7	43.8 ± 2.4
50-59 years	*n*	65	22
	Age	55.0 ± 2.9	56.3 ± 2.4
60-69 years	*n*	114	61
	Age	64.4 ± 2.6	64.7 ± 2.9
> 69 years	*n*	47	29
	Age	74.9 ± 4.5	73.4 ± 3.6
Total	*n*	247	129
	Age	62.3 ± 8.9	62.5 ± 9.5

**Table 4:** Mean and standard deviation (SD) of the six analyzed texture features and vBMD of females, grouped by spine region.

Spine region (females)		T4-T8	T9-T12	L1-L5
Variance_global_	Mean	24.60	39.24	55.94
	SD	5.30	6.24	6.62
Entropy	Mean	14.16	14.40	14.60
	SD	0.59	0.66	0.73
SRE	Mean	0.9922	0.9916	0.9916
	SD	0.0023	0.0025	0.0025
LRE	Mean	1.0322	1.0344	1.0344
	SD	0.0097	0.0105	0.0106
RLN	Mean	0.9794	0.9780	0.9781
	SD	0.0060	0.0064	0.0065
RP	Mean	0.9895	0.9888	0.9888
	SD	0.0031	0.0033	0.0034
vBMD [mg/cm³]	Mean	137.2	131.5	128.9
	SD	39.8	39.4	39.3

SRE, short-run emphasis; LRE, long-run emphasis; RLN, run-length non-uniformity; RP, run percentage; vBMD, volumetric bone mineral density.

**Table 5:** Mean and standard deviation (SD) of the six analyzed texture features and vBMD of males, grouped by spine region.

Spine region (males)		T4-T8	T9-T12	L1-L5
Variance_global_	Mean	32.42	51.21	68.03
	SD	7.02	8.21	7.80
Entropy	Mean	14.27	14.34	14.47
	SD	0.57	0.63	0.73
SRE	Mean	0.9914	0.9905	0.9909
	SD	0.0022	0.0025	0.0026
LRE	Mean	1.0354	1.0392	1.0377
	SD	0.0095	0.0109	0.0110
RLN	Mean	0.9774	0.9751	0.9760
	SD	0.0058	0.0066	0.0067
RP	Mean	0.9885	0.9873	0.9878
	SD	0.0030	0.0034	0.0035
vBMD [mg/cm³]	Mean	144.2	133.3	127.7
	SD	36.1	34.1	33.5

SRE, short-run emphasis; LRE, long-run emphasis; RLN, run-length non-uniformity; RP, run percentage; vBMD, volumetric bone mineral density.

**Table 6:** Mean and standard deviation (SD) of the six analyzed texture features and vBMD of females, grouped by age group.

Age group (females)		<50	50-59	60-69	≥70
Variance_global_	Mean	38.06	41.79	39.60	38.68
	SD	14.66	15.02	14.59	13.42
Entropy	Mean	14.54	14.42	14.28	14.47
	SD	0.48	0.68	0.68	0.78
SRE	Mean	0.9923	0.9918	0.9915	0.9922
	SD	0.0019	0.0024	0.0024	0.0027
LRE	Mean	1.0317	1.0335	1.0349	1.0320
	SD	0.0080	0.0101	0.0104	0.0114
RLN	Mean	0.9797	0.9786	0.9778	0.9795
	SD	0.0049	0.0062	0.0063	0.0070
RP	Mean	0.9897	0.9891	0.9887	0.9896
	SD	0.0025	0.0032	0.0033	0.0036
vBMD [mg/cm³]	Mean	179.6	134.0	126.0	113.7
	SD	43.7	33.5	33.7	26.4

SRE, short-run emphasis; LRE, long-run emphasis; RLN, run-length non-uniformity; RP, run percentage; vBMD, volumetric bone mineral density.

**Table 7:** Mean and standard deviation (SD) of the six analyzed texture features and vBMD of males, grouped by age group.

Age group (males)		<50	50-59	60-69	≥70
Variance_global_	Mean	49.65	50.12	50.69	51.26
	SD	17.06	16.86	16.81	16.77
Entropy	Mean	14.31	14.33	14.39	14.36
	SD	0.49	0.65	0.68	0.66
SRE	Mean	0.9909	0.9908	0.9911	0.9907
	SD	0.0020	0.0025	0.0025	0.0026
LRE	Mean	1.0373	1.0379	1.0366	1.0383
	SD	0.0086	0.0109	0.0104	0.0112
RLN	Mean	0.9762	0.9759	0.9767	0.9757
	SD	0.0052	0.0065	0.0064	0.0068
RP	Mean	0.9879	0.9877	0.9881	0.9876
	SD	0.0027	0.0034	0.0033	0.0035
vBMD [mg/cm³]	Mean	154.1	142.2	131.1	125.2
	SD	38.1	37.2	33.4	29.8

SRE, short-run emphasis; LRE, long-run emphasis; RLN, run-length non-uniformity; RP, run percentage; vBMD, volumetric bone mineral density.

All of the six analyzed TFs, except Entropy, showed a statistically significant difference between genders (**Table 8**).

**Table 8:** Texture features and vBMD, averaged from T4-L5 and grouped by gender.

	Male	Female	p-value
Variance_global_	50.54 ± 16.84	39.56 ± 14.48	<0.001
Entropy	14.36 ± 0.65	14.38 ± 0.68	n.s.
SRE	0.9910 ± 0.0025	0.9918 ± 0.0024	<0.001
LRE	1.0373 ± 0.0106	1.0336 ± 0.0103	<0.001
RLN	0.9763 ± 0.0064	0.9785 ± 0.0063	<0.001
RP	0.9879 ± 0.0033	0.9891 ± 0.0033	<0.001
vBMD [mg/cm³]	135.2 ± 35.3	132.7 ± 39.6	0.029

Bonferroni-corrected p-values indicate statistically significant differences between genders. SRE, short-run emphasis; LRE, long-run emphasis; RLN, run-length non-uniformity; RP, run percentage; n.s., not significant.

**Figure 3:**
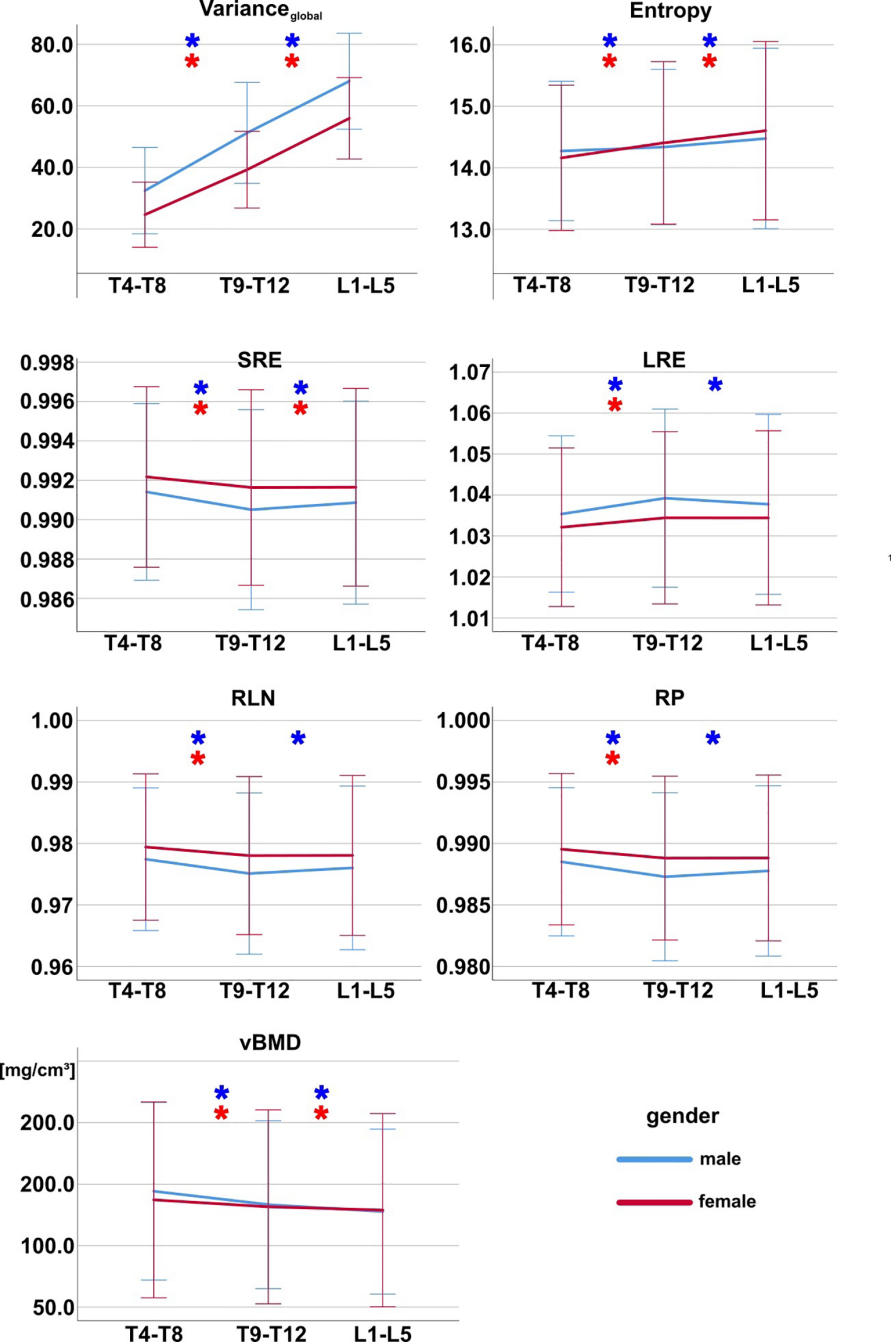
Texture features and vBMD grouped by spine region for females (red) and males (blue). Asterisks (*) mark pairwise significant differences between spine regions. Bonferroni correction was applied to adjust for multiple comparisons. Error bars indicate ± 2 standard deviations. SRE, short-run emphasis; LRE, long-run emphasis; RLN, run-length non-uniformity; RP, run percentage; vBMD, volumetric bone mineral density.

Variance_global_ and Entropy showed a statistically significant increase in cranio-caudal direction along the spine across all subjects as well as for both genders separately (**Figure 3** and **Tables 4**, **5**). All other TFs (SRE, LRE, RLN and RP) showed statistically significant differences between spine regions across all subjects (**Table 9**). SRE, LRE, RLN and RP showed also statistically significant differences between spine regions for both genders separately, except for LRE, RLN and RP between T9-T12 and L1-L5 in females (**Figure 3** and **Tables 4**, **5**).

**Table 9:** Pairwise significant differences of texture features between spine regions across all subjects.

Texture feature	T4-T8 vs T9-T12	T4-T8 vs L1-L5	T9-T12 vs L1-L5
Variance_global_	<0.001	<0.001	<0.001
Entropy	<0.001	<0.001	<0.001
SRE	<0.001	<0.001	0.036
LRE	<0.001	<0.001	0.041
RLN	<0.001	<0.001	0.036
RP	<0.001	<0.001	0.038

Bonferroni-corrected p-values corresponding to a level of significance of 0.05 are given. SRE, short-run emphasis; LRE, long-run emphasis; RLN, run-length non-uniformity; RP, run percentage.

**Figure 4:**
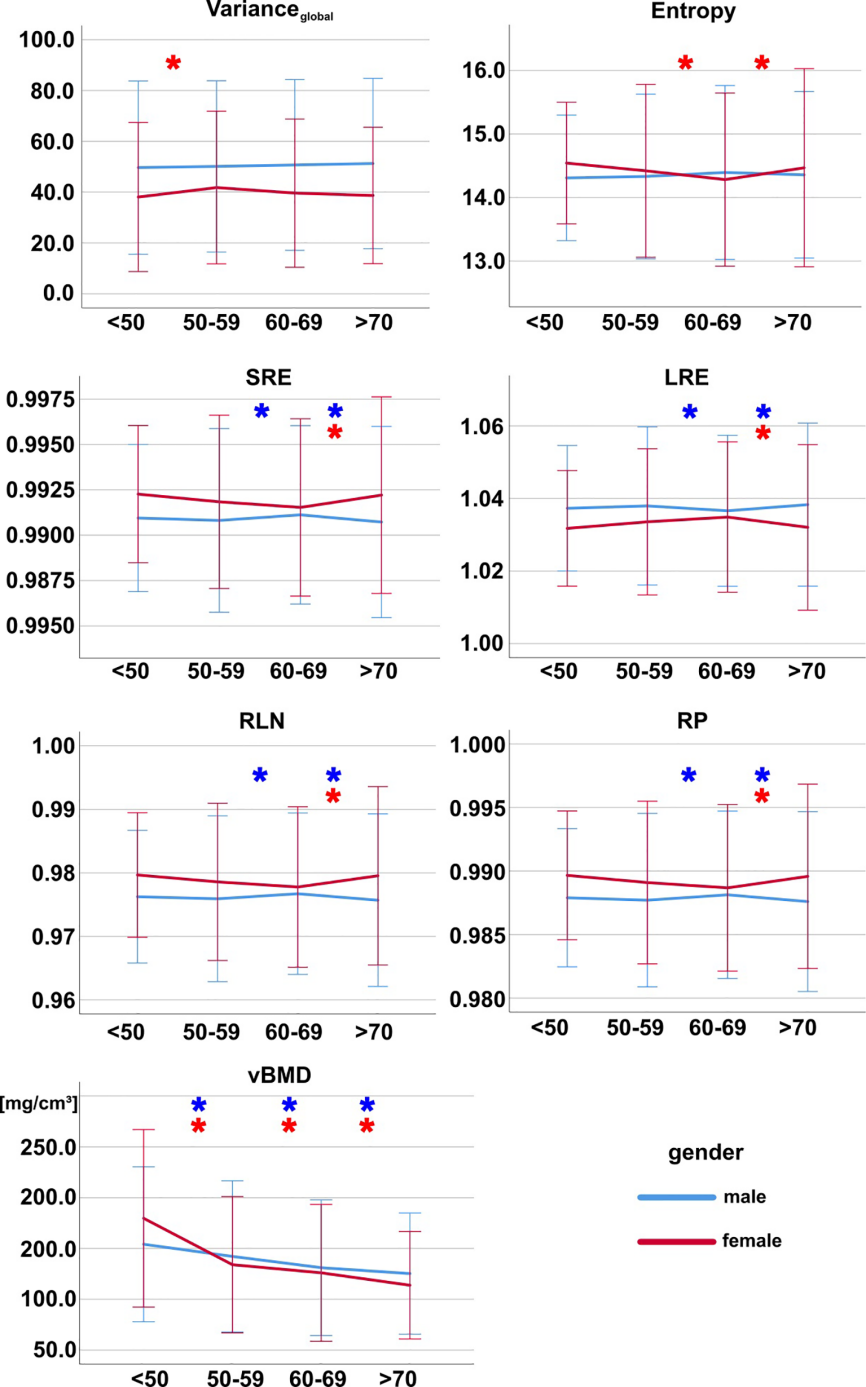
Texture features and vBMD grouped by age group for females (red) and males (blue). Asterisks (*) mark pairwise significant differences between consecutive age groups. Bonferroni correction was applied to adjust for multiple comparisons. Error bars indicate ± 2 standard deviations. SRE, short-run emphasis; LRE, long-run emphasis; RLN, run-length non-uniformity; RP, run percentage; vBMD, volumetric bone mineral density.

For both males and females, certain TFs showed a statistically significant difference between age groups, however without a clear directional trend (**Figure 4** and **Tables 6**, **7**).

vBMD showed a statistically significant difference between genders (**Table 8**), a statistically significant decrease with age as well as statistically significant differences between spine regions for both males and females (**Figures 3**, **4**).

Linear correlation analysis revealed statistically significant (p < 0.05) low positive associations (*r_vBMD,Entropy_* = 0.39, *r_vBMD,SRE_* = 0.31, *r_vBMD,RLN_* = 0.31, *r_vBMD,RP_* = 0.31), and very low to low negative associations (*r_vBMD,Varianceglobal_* = -0.19, *r_vBMD,LRE_* = -0.31) between the analyzed TFs and vBMD, respectively.

## Discussion

The present study performed automated spine segmentation and TF extraction of vertebral bodies in routine abdominal MDCT scans in a large-scale patient cohort. In a preceding analysis, long-term reproducibility of a set of commonly utilized TFs was evaluated. Six TFs with high reproducibility were identified and selected for further analysis. To the best of our knowledge, this is the first study combining fully automated segmentation, vBMD mapping and long-term reproducibility analysis to establish gender-, age-, and region-specific characteristic values and variations of vertebral TFs. This data could serve as foundation for a reliable differentiation of values indicative of osteoporosis from normal values across genders, age groups and spine regions. In particular, it could be a prerequisite for the longitudinal comparison of TFs.

Variance_global_ and Entropy showed a significant increase in cranio-caudal direction along the spine for both males and females. The other four TFs, which are all GLRLM-derived features, also showed significant region-dependent differences, except for females between the lower thoracic and lumbar spine. Five of the six TFs showed a significant difference between genders. None of the TFs showed a significant age-dependence. vBMD showed a significant decrease with age as well as statistically significant differences between genders and spine regions, confirming results from previous studies (13, 18, 29).

Dimension reduction and feature selection is a commonly performed step in TA studies to reduce redundancy, and to ensure that selected features fulfill certain conditions and have high relevance. However, the applied approaches are manifold and vary substantially between studies. Among many other methods, GLCM- and GLRLM-derived features can be restricted to or averaged across directions and distances (7, 30). Another popular approach is to only select reproducible features with high inter- and intra-reader agreement (8, 30). Mannil et al. additionally performed correlation analysis and excluded highly correlating TFs to reduce redundancy (30). The emergence of machine learning has further extended the repertoire of feature reduction methods. Using a random forest classifier to identify vertebral fractures, Valentinitsch et al. optimized the number of features based on the Gini importance and classification performance in an exponential search (9). We chose a more clinically driven approach and selected TFs based on a preceding long-term reproducibility analysis to identify features particularly suited for longitudinal comparisons.

Our study determined characteristic TF values based on CT images of vertebral bodies. The found values for Variance_global_ show substantial differences to the values reported by Mannil et al. (30), who performed the only similar study we could find in previous literature. This discrepancy, however, is potentially due to the limited comparability of the two studies. While Mannil et al. used manually prescribed ROIs of mid-sagittal planes of the vertebral body for two-dimensional (2D) TA (30), we utilized 3D-ROIs of the entire volume of the trabecular compartment of the vertebral body with subsequent 3D-TA. As a result, number and intra-vertebral localization of the included voxels as well as their gray value distribution vary remarkably between the two studies. Differences in CT image acquisition and reconstruction as well as slice thickness (2 mm *vs*. 3 mm) may additionally contribute to the discrepancy of the reported TF values. Fully automated labeling and 3D-segmentation of the spine represent major improvements in terms of time efficiency, reproducibility and utilization of available CT data when compared to manually performed 2D-segmentations. Therefore, the segmentation approach used in the present study should be considered as the standard for TA of the spine.

Characteristic values of TFs should be used and compared with great care and ideally only if acquisition and reconstruction parameters as well as postprocessing methods are identical. Therefore, we used images acquired at the same MDCT scanner with a standardized protocol, a fully automated and standardized segmentation method, as well as careful preparation of CT data prior to TF computation, including isotropic resampling and gray-level normalization.

The TFs Variance_global_ and Entropy showed significant regional differences, increasing from the mid-thoracic to the lumbar spine, irrespective of gender. Along the spine, more inferior vertebral bodies have larger size as well as a higher prevalence and degree of degenerative changes (29, 31). This could be potential explanations for the cranio-caudal increase in Variance_global_ since it reflects the spread of gray-level distribution. The GLCM feature Entropy is a measure of randomness in an image. However, it is not trivial to define what image properties it actually represents. According to Haralick et al., Entropy is associated with gray-level number, complexity and degree of structure in an image (21, 32). Therefore, the found region-dependent variations could indicate a gradient in architectural complexity along the spine, potentially suggesting variability in trabecular microstructure and skeletal integrity (33).

The TFs SRE, LRE, RP and RLN also showed gender-specific differences, as well as region-dependent differences, irrespective of gender for most features. These GLRLM features are highly associated with each other and, according to their defining equations, reflect the predominating length of gray-level runs, the distribution of gray-level run lengths, as well as the extent of linear structures (22). The variations we found may therefore indicate gender- and region-specific variability in trabecular microstructure, in line with previous ex vivo results (34).

There are several limitations to our study. First, we performed a retrospective analysis with limited availability of weight and height as well as other bone health-related data of the study cohort. Therefore, we did not adjust our results for variations in body mass index (BMI) or other potential confounders, such as smoking status, which are known to be correlated with BMD and could also have an influence on TFs. Second, n = 376 is relatively small sample size for establishing reference values, and the age range of the study is limited, in particular the age group under 40 years is not represented in our cohort. However, primary osteoporosis, as the most important condition for clinical application of the herein used approach and derived characteristic TF values, usually does not start before the age of 40 years. In contrast to values derived from a population of young individuals with normal bone health, age-, gender- and region-specific may also represent a viable approach for the differentiation of physiological and pathological bone alterations of the spine. Nevertheless, extending the analysis to an increased study population with a broader age range should be an aim of future investigations, in order to firmly establish a reference database. Third, the initial set of TFs we considered in our analysis was limited, and additional TFs (e.g. based on local binary patterns, wavelets or other transforms) could have been used (35, 36). Fourth, to maximize comparability of the computed values, CT data acquisition and postprocessing as well as TA were performed with homogenous and standardized settings. The restriction to a specific CT scanner and acquisition protocol may reduce the utility of the findings to other investigations using different settings. However, TF values, in particular for GLCM- and GLRLM-derived features, can only be reliably used for comparisons in studies with identical settings.

## Conclusion

In conclusion, we established highly reproducible TF values for CT-based 3D-TA of vertebral bodies in a predominantly older study population, using an automated segmentation and quantification pipeline. These characteristic TF values and the found gender-, age-, and vertebral-level-specific variations can be considered a foundation for the reliable differentiation of physiological and pathological alterations and may be particularly important for future longitudinal studies.

## Data Availability Statement

The raw data supporting the conclusions of this article will be made available by the authors, without undue reservation.

## Ethics Statement

The studies involving human participants were reviewed and approved by Ethikkommission der Technischen Universität München. Written informed consent for participation was not required for this study in accordance with the national legislation and the institutional requirements.

## Author Contributions

MD wrote the manuscript. MD organized the database. MD performed the formal and statistical analysis. MD contributed to the concept and design of the study. MD developed major parts of the used software routines. NS, MH, AS, ML, CZ, JK, KS, and TB critically revised the manuscript. MH, AS, and ML contributed to the development of the software used in the study. TB conceptualized and designed the study. CZ, JK, KS, and TB provided scientific supervision and resources. JK and TB provided funding. TB contributed to the generation and organization of the database. All authors contributed to the article and approved the submitted version.

## Funding

The present work was supported by the German Research Foundation (Deutsche Forschungsgemeinschaft, DFG, project 432290010, JK and TB), the German Society of Musculoskeletal Radiology (Deutsche Gesellschaft für Muskuloskelettale Radiologie, DGMSR, MD and NS), and the B. Braun Foundation (project BBST-D-19-00106, NS).

## Conflict of Interest

The authors declare that the research was conducted in the absence of any commercial or financial relationships that could be construed as a potential conflict of interest.

## Publisher’s Note

All claims expressed in this article are solely those of the authors and do not necessarily represent those of their affiliated organizations, or those of the publisher, the editors and the reviewers. Any product that may be evaluated in this article, or claim that may be made by its manufacturer, is not guaranteed or endorsed by the publisher.

## Abbreviations

3D, Three-dimensional; BL, Baseline; BMD, Bone mineral density; CT, Computed tomography; FU, Follow-up; GLCM, Gray-level co-occurrence matrix; GLN, Gray-level non-uniformity; GLRLM, Gray-level run-length matrix; GLV, Gray-level variance; HGLRE, High gray-level run emphasis; HU, Hounsfield units; IVCM, Intravenous contrast medium; LGLRE, Low gray-level run emphasis; LRE, Long-run emphasis; MRI, Magnetic resonance imaging; LRHGLE, Long-run high gray-level emphasis; LRLGLE, Long-run low gray-level emphasis; MDCT, Multi-detector computed tomography; PACS, Picture archiving and communication system; QCT, Quantitative computed tomography; *r_vBMD,TF,_* Pearson correlation coefficient of vBMD and texture feature (*TF* = Variance_global_, Entropy, SRE, LRE, RLN, RP); ROI, Region of interest; RLN, Run-length non-uniformity; RLV, Run-length variance; RP, Run percentage; SD, Standard deviation; SRE, Short-run emphasis; SRHGLE, Short-run high gray-level emphasis; SRLGLE, Short-run low gray-level emphasis; TA, Texture analysis; TF, Texture feature; vBMD, Volumetric bone mineral density; VF, Vertebral fracture; Δ_rel,_ Reproducibility error (relative difference between BL and FU measurement).

## References

[1] Choi JY. Radiomics and Deep Learning in Clinical Imaging: What Should We do? Nucl Med Mol Imaging (2018) 52(2):89–90. doi: 10.1007/s13139-018-0514-0 29662556 PMC5897263

[2] Davnall F Yip CS Ljungqvist G Selmi M Ng F Sanghera B. Assessment of Tumor Heterogeneity: An Emerging Imaging Tool for Clinical Practice? Insights Imaging (2012) 3(6):573–89. doi: 10.1007/s13244-012-0196-6 PMC3505569 23093486

[3] O'Connor JP Rose CJ Waterton JC Carano RA Parker GJ Jackson A. Imaging Intratumor Heterogeneity: Role in Therapy Response, Resistance, and Clinical Outcome. Clin Cancer Res (2015) 21(2):249–57. doi: 10.1158/1078-0432.CCR-14-0990 PMC4688961 25421725

[4] Lubner MG Smith AD Sandrasegaran K Sahani DV Pickhardt PJ. CT Texture Analysis: Definitions, Applications, Biologic Correlates, and Challenges. Radiographics (2017) 37(5):1483–503. doi: 10.1148/rg.2017170056 28898189

[5] Mannil M Burgstaller JM Held U Farshad M Guggenberger R. Correlation of Texture Analysis of Paraspinal Musculature on MRI With Different Clinical Endpoints: Lumbar Stenosis Outcome Study (LSOS). Eur Radiol (2019) 29(1):22–30. doi: 10.1007/s00330-018-5552-6 29948080

[6] Dieckmeyer M Junker D Ruschke S Mookiah MRK Subburaj K Burian E. Vertebral Bone Marrow Heterogeneity Using Texture Analysis of Chemical Shift Encoding-Based MRI: Variations in Age, Sex, and Anatomical Location. Front Endocrinol (Lausanne) (2020) 11:555931. doi: 10.3389/fendo.2020.555931 33178134 PMC7593641

[7] Mookiah MRK Rohrmeier A Dieckmeyer M Mei K Kopp FK Noel PB. Feasibility of Opportunistic Osteoporosis Screening in Routine Contrast-Enhanced Multi Detector Computed Tomography (MDCT) Using Texture Analysis. Osteoporos Int (2018) 29(4):825–35. doi: 10.1007/s00198-017-4342-3 29322221

[8] Muehlematter UJ Mannil M Becker AS Vokinger KN Finkenstaedt T Osterhoff G. Vertebral Body Insufficiency Fractures: Detection of Vertebrae at Risk on Standard CT Images Using Texture Analysis and Machine Learning. Eur Radiol (2019) 29(5):2207–17. doi: 10.1007/s00330-018-5846-8 30519934

[9] Valentinitsch A Trebeschi S Kaesmacher J Lorenz C Loffler MT Zimmer C. Opportunistic Osteoporosis Screening in Multi-Detector CT Images *via* local classification of textures. Osteoporos Int (2019) 30(6):1275–85. doi: 10.1007/s00198-019-04910-1 PMC6546649 30830261

[10] Consensus Development Conference: Diagnosis, Prophylaxis, and Treatment of Osteoporosis. Am J Med (1993) 94(6):646–50. doi: 10.1016/0002-9343(93)90218-e 8506892

[11] Drake MT Clarke BL Lewiecki EM. The Pathophysiology and Treatment of Osteoporosis. Clin Ther (2015) 37(8):1837–50. doi: 10.1016/j.clinthera.2015.06.006 26163201

[12] Link TM. Osteoporosis Imaging: State of the Art and Advanced Imaging. Radiology (2012) 263(1):3–17. doi: 10.1148/radiol.12110462 22438439 PMC3309802

[13] Loffler MT Sollmann N Mei K Valentinitsch A Noel PB Kirschke JS. X-Ray-Based Quantitative Osteoporosis Imaging at the Spine. Osteoporos Int (2020) 31(2):233–50. doi: 10.1007/s00198-019-05212-2 31728606

[14] Sekuboyina A Bayat A Löffler M Liebl H Li H. VerSe: A Vertebrae Labelling and Segmentation Benchmark for Multi-Detector CT Images. Med Image Anal (2021) 73:102166. doi: 10.1016/j.media.2021.102166 34340104

[15] Loffler MT Sekuboyina A Jacob A Grau AL Scharr A El Husseini M. A Vertebral Segmentation Dataset With Fracture Grading. Radiol Artif Intell (2020) 2(4):e190138. doi: 10.1148/ryai.2020190138 33937831 PMC8082364

[16] Sekuboyina A Husseini ME Bayat A Loffler M Liebl H Li H. Verse: A Vertebrae Labelling and Segmentation Benchmark for Multi-Detector CT Images. Med Image Anal (2021) 73:102166. doi: 10.1016/j.media.2021.102166 34340104

[17] Radiology ACo. ACR–SPR–SSR Practice Parameter for the Performance of Musculoskeletal Quantitative Computed Tomography (QCT)(2018). Available at: https://www.acr.org/-/media/ACR/Files/Practice-Parameters/QCT.pdf?la=en.

[18] Link TM Lang TF. Axial QCT: Clinical Applications and New Developments. J Clin Densitom (2014) 17(4):438–48. doi: 10.1016/j.jocd.2014.04.119 24880494

[19] Mookiah MRK Baum T Mei K Kopp FK Kaissis G Foehr P. Effect of Radiation Dose Reduction on Texture Measures of Trabecular Bone Microstructure: An *In Vitro* Study. J Bone Miner Metab (2018) 36(3):323–35. doi: 10.1007/s00774-017-0836-5 28389933

[20] Gaztañaga E Croft R Dalton G. Variance, Skewness & Kurtosis: Results From the APM Cluster Redshift Survey and Model Predictions. Mon Not R Astron Soc (1995) 276:336–45. doi: 10.1093/mnras/276.1.336

[21] Haralick RM Shanmugam K Dinstein I. Textural Features for Image Classification. IEEE Trans Syst Man Cybern (1973) SMC-3(6):610–21. doi: 10.1109/TSMC.1973.4309314

[22] Galloway MM. Texture Analysis Using Gray Level Run Lengths. Comput Graphics Image Process (1975) 4(2):172–9. doi: 10.1016/S0146-664X(75)80008-6

[23] Chu A Sehgal C Greenleaf J. Use of Gray Value Distribution of Run Lengths for Texture Analysis. Pattern Recognit Lett (1990) 11:415–9. doi: 10.1016/0167-8655(90)90112-F

[24] Dasarathy BV Holder EB. Image Characterizations Based on Joint Gray Level—Run Length Distributions. Pattern Recognit Lett (1991) 12(8):497–502. doi: 10.1016/0167-8655(91)80014-2

[25] Thibault G Fertil B Navarro C Pereira S Cau P Lévy N. Texture Indexes and Gray Level Size Zone Matrix. Application to Cell Nuclei Classification. 10th International Conference on Pattern Recognition and Information Processing (2009).

[26] Nailon WH. Texture Analysis Methods for Medical Image Characterisation. In: Mao Y, editor. Biomedical Imaging. London, United Kingdom: InTech (2010). p. 75–100. doi: 10.5772/205

[27] Vallieres M Freeman CR Skamene SR El Naqa I. A Radiomics Model From Joint FDG-PET and MRI Texture Features for the Prediction of Lung Metastases in Soft-Tissue Sarcomas of the Extremities. Phys Med Biol (2015) 60(14):5471–96. doi: 10.1088/0031-9155/60/14/5471 26119045

[28] Kaesmacher J Liebl H Baum T Kirschke JS. Bone Mineral Density Estimations From Routine Multidetector Computed Tomography: A Comparative Study of Contrast and Calibration Effects. J Comput Assist Tomogr (2017) 41(2):217–23. doi: 10.1097/RCT.0000000000000518 PMC5359785 27798444

[29] Valentinitsch A Trebeschi S Alarcon E Baum T Kaesmacher J Zimmer C. Regional Analysis of Age-Related Local Bone Loss in the Spine of a Healthy Population Using 3D Voxel-Based Modeling. Bone (2017) 103:233–40. doi: 10.1016/j.bone.2017.06.013 28716553

[30] Mannil M Eberhard M Becker AS Schonenberg D Osterhoff G Frey DP. Normative Values for CT-Based Texture Analysis of Vertebral Bodies in Dual X-Ray Absorptiometry-Confirmed, Normally Mineralized Subjects. Skeletal Radiol (2017) 46(11):1541–51. doi: 10.1007/s00256-017-2728-0 28780746

[31] Leboeuf-Yde C Nielsen J Kyvik KO Fejer R Hartvigsen J. Pain in the Lumbar, Thoracic or Cervical Regions: Do Age and Gender Matter? A Population-Based Study of 34,902 Danish Twins 20-71 Years of Age. BMC Musculoskelet Disord (2009) 10:39. doi: 10.1186/1471-2474-10-39 19379477 PMC2678974

[32] Haralick R Anderson DE. Texture-Tone Study With Application to Digitized Imagery CRES Technical Report 182-2. Lawrence, Kansas: The University of Kansas Center for Research, Inc. (1971).

[33] Tabari A Torriani M Miller KK Klibanski A Kalra MK Bredella MA. Anorexia Nervosa: Analysis of Trabecular Texture With CT. Radiology (2017) 283(1):178–85. doi: 10.1148/radiol.2016160970 PMC5375622 27797678

[34] Yeni YN Zinno MJ Yerramshetty JS Zauel R Fyhrie DP. Variability of Trabecular Microstructure is Age-, Gender-, Race- and Anatomic Site-Dependent and Affects Stiffness and Stress Distribution Properties of Human Vertebral Cancellous Bone. Bone (2011) 49(4):886–94. doi: 10.1016/j.bone.2011.07.006 PMC3170516 21802536

[35] Ojala T Pietikainen M Maenpaa T. Multiresolution Gray-Scale and Rotation Invariant Texture Classification With Local Binary Patterns. IEEE Trans Pattern Anal Mach Intell (2002) 24(7):971–87. doi: 10.1109/TPAMI.2002.1017623

[36] Ngo V Dinh TN. Bone Texture Characterization Based on Contourlet and Gabor Tranforms. Int J Comput Theory Eng (2016) 8:177–81. doi: 10.7763/IJCTE.2016.V8.1040

